# Knowledge about Sugar Sources and Sugar Intake Guidelines in Portuguese Consumers

**DOI:** 10.3390/nu12123888

**Published:** 2020-12-19

**Authors:** Marília Prada, Magda Saraiva, Margarida V. Garrido, David L. Rodrigues, Diniz Lopes

**Affiliations:** Department of Social and Organizational Psychology, Iscte-Instituto Universitário de Lisboa, CIS_Iscte, Av. das Forças Armadas, Office AA110, 1649-026 Lisboa, Portugal; magda.saraiva@iscte-iul.pt (M.S.); margarida.garrido@iscte-iul.pt (M.V.G.); dflrs@iscte-iul.pt (D.L.R.); diniz.lopes@iscte-iul.pt (D.L.)

**Keywords:** free sugars, food policy, knowledge, guidelines, sugar sources, sugar intake

## Abstract

In this work, we examined knowledge about sugars and guidelines for its consumption and explored the relationship between knowledge and measures related to nutritional information processing as well as sugar consumption. Specifically, we asked participants (*n* = 1010 Portuguese) to categorize a set of ingredients (e.g., glucose, aspartame) regarding their composition (i.e., intrinsic vs. added/free sugars) and origin (e.g., natural vs. artificial) and if they were aware of the WHO guidelines for sugar intake. Overall, despite using information about sugar frequently and considering attending to such information as very important to stay healthy, most participants were unaware of the WHO guidelines and revealed difficulties in the categorization task. Women, participants with a higher level of education, and those with children in the household reported higher use of sugar content information present in nutritional labels, higher perceived knowledge of nutritional guidelines, and higher hit rates in categorizing sugar sources. Almost one-fourth of the population exceeds the daily limit recommended by the WHO. Therefore, characterizing the knowledge of a Portuguese sample regarding sugar sources and sugar intake guidelines is particularly relevant, and our results emphasize the need to implement effective strategies to reduce sugar intake.

## 1. Introduction

Currently, inadequate dietary habits constitute a major health concern as they contribute to obesity and increased risk of chronic non-communicable diseases such as diabetes or cardiovascular and respiratory diseases [1]. Obesity, in particular, constitutes a serious public health concern [2] and is currently considered a global epidemic [1], affecting all age groups. In addition, obesity is associated with an increased risk of death [3,4,5]. For example, obese individuals are likely to die 9.44 years earlier than individuals of reference weight [6].

Excessive sugar intake is strongly associated with obesity, as it contributes to an increase in total energy consumption, usually without other nutritional benefits (e.g., vitamins or minerals), and it is converted into fat and stored in the body (for a review see [7]). Moreover, the consumption of high sugar drinks and foods also appears to be associated with an increased risk of cancer [8,9]. Therefore, any measure that seeks to reduce sugar intake may also contribute to reduce the incidence of non-communicable diseases [1].

A recent study, including data from 11 European countries, showed that total sugar intake is mostly determined by the consumption of sweets products such as chocolates, cakes, and biscuits [10]. These products are typically high in free sugars that have particularly adverse effects on health (for a review, see [11]). According to the WHO [1,12], free sugars include all the monosaccharides and disaccharides added to foods by the manufacturer or consumer, and all the sugars that are naturally present in honey, syrups, fruit juices, and fruit concentrates. 

The WHO [1] recommends reducing free sugars intake throughout the life-course. Specifically, both children and adults should limit the consumption of free sugars to less than 10% of total energy consumption, ideally below 5% of their total energy intake. A general strategy that has been used to reduce sugar intake is to increase consumers’ knowledge about sugar [13], based on the assumption that knowledge can change attitudes and consequently behaviors [14]. Some studies have supported this assumption reporting an association between increasing knowledge about sugar and reduced consumption of food and beverages with sugar [15,16,17]. Nevertheless, research focused on consumers’ knowledge of sugars suggests the existence of misconceptions regarding sugar sources and a generalized lack of knowledge about sugar intake guidelines. 

Low consumer knowledge of sugar sources is evident in studies using different tasks, including the categorization (or estimation) of sugar content in products and the recognition of ingredients as sugar sources. For example, the authors in [18] asked a sample of 3361 American consumers to categorize a set of beverages as “without sugar”, “with natural sugar”, “with added sugar”, or “with artificial sweetener”. Even though most consumers (78%) were able to identify that regular soft drinks contain added sugar, only 58% could do so for sports drinks. The accuracy in identifying natural sugars was even lower, with 54% and 23% of participants correctly categorizing 100% vegetable juice and milk as containing natural sugars, respectively. Likewise, a recent study with 2732 Australian consumers showed that only 34% of the participants correctly estimated the sugar content of soft drinks [19].

On the other hand, consumers also struggle with the identification of certain ingredients as sugar sources. For example, Tierney et al. [20] asked 445 Northern Irish participants to categorize a list of 13 food ingredients as natural (i.e., intrinsic) sugar, as added sugar, or as artificial sweeteners. Results showed that participants were often unable to answer or miscategorized added sugars as intrinsic ones (e.g., almost 90% of participants categorized honey as a natural sugar). Importantly, these results do not seem to stem from a general lack of interest in nutrition or food sugar content. Indeed, participants reported that sugar content is one of the items most frequently attended in the nutritional facts panel and one of the most important items to consider in order to stay healthy.

Results regarding the knowledge of free sugars intake guidelines are also worrisome. For example, Vanderlee et al. [21] asked participants to identify the WHO guidelines regarding the consumption of added and total sugars. Results revealed that only 7.5% of participants (*n* = 2008 Canadians, 16–24 years old) were able to identify the 10% or 5% recommendations. Likewise, findings from another study [20] showed that 65% of the participants reported being unaware of the WHO’s guidelines about free sugars intake. 

In light of these alarming findings, it is highly relevant to explore the factors associated with the low reported knowledge about sugar sources and guidelines. While most processed foods include added sugars [22,23], the actual added or free sugar content is rarely presented in nutritional labels. For example, in the European Union (EU), regulations on the provision of food information to consumers state that the “mandatory nutrition declaration shall include the following: (a) energy value; and the amounts of (b) fat, saturates, carbohydrate, sugars, protein and salt” (Regulation EU No 1169/2011; Article 30). Hence, a consumer interested in determining which proportion of the total sugars described in the nutrition information panel constitutes added sugar would have to consider the ingredient list. Still, that may prove to be a challenging task due to the multiple types of sugar that exist. For instance, Bernstein et al. [24] identified over 150 types of free sugars ingredients, including sucrose, either dried/granulated (e.g., sugar, cane sugar, beet sugar) or in syrups (e.g., invert sugar syrup, caramel, treacle), glucose (e.g., glucose solids, dextrose, dextrin syrup), or molasses (e.g., black molasses, cane juice molasses, cooking molasses). As illustrated by these examples, some ingredients may be quite hard to recognize. 

Previous research has also shown that consumers often infer healthiness based on other food proprieties (e.g., gluten-free, [25]), and this may also constitute a potential source of bias regarding the identification of free sugars. For example, people tend to perceive an association between naturalness and healthiness (for a review, see [26]). To illustrate, fruit nectars and juices, in comparison with sodas, are often perceived as healthier [27] and as containing less sugar [19]. Another study showed that cereals with “fruit sugar” were perceived as healthier than those labeled as having “sugar” [28]. These results suggest that participants may be inferring that a sugar of natural origin (i.e., fruit) is also an intrinsic sugar, regardless of being added to a product (i.e., breakfast cereals). Indeed, when asked to categorize a list of ingredients, the majority of participants in Tierney’s et al. study [20] miscategorized “fruit juice” and “fructose” as natural sugars (around 70% and 60%, respectively) and, in the case of “honey”, the error rate was almost 90%.

### The Current Study

To the best of our knowledge, previous research examining knowledge about sugar has not included a Portuguese sample. In Portugal, the prevalence of overweight is 55.6%, and obesity is 20.1% [29] and inadequate eating habits are estimated to contribute to the loss of 15.4% of healthy years of life [30]. Furthermore, 24.3% of the adult Portuguese population exceeds the recommended sugar intake, and this prevalence is particularly worrying in adolescents (48.7%) and children (40.7%), [29]. These high numbers of sugar intake result, for example, from a daily consumption of one or more soft drinks or fruit nectars: varying from 3% in older adults up to 42% in adolescents (18% in the overall Portuguese population, [29]). Acknowledging the WHO guidelines, Portuguese health authorities have been implementing strategies to reduce sugar consumption (for a review, see [31]). For example, a recent law introduced restrictions on foodstuffs advertising containing high energy value, salt, sugar, and saturated and processed fatty acids (Law No. 30/2019, Diário da República, 23 April 2019). Other measures included limiting unhealthy products in vending machines available in institutions under the Ministry of Health (Order no. 7516-A/2016, Diário da República), and increasing taxes on beverages with high sugar content (Order no. 42/2016, Diário da República). 

Considering the negative impacts of excessive sugar consumption and the role that individual knowledge can play in changing eating habits, the current study aims to characterize knowledge about sugar sources and the available guidelines for sugar intake in a sample of Portuguese consumers. Additionally, we will also examine whether knowledge is associated with other variables related to the interest in and ability to process nutritional information (e.g., frequency of use of nutritional information; food literacy), which are likely to influence eating behavior, particularly sugar intake. 

## 2. Materials and Methods

### 2.1. Participants

A sample of 1010 Portuguese individuals (76.7% women, 22.9% men, and 0.4% other), with ages between 18 and 82 years (*M* = 36.33, *SD* = 13.22), volunteered to participate in this survey. Almost half of the participants reported having a college degree (44.9%) and being employed (77.5%). Most participants also indicated they were in a romantic relationship, either cohabitating with the partner (50.0%) or living apart (22.1%), had no children in the household (66.5%), and were the main person responsible for household shopping (51.8%). Most participants reported following a regular omnivorous diet (72.5%), with a BMI within the normal (58.5%) or overweight (24.1%) weight range.

### 2.2. Procedure and Measures

This study was part of a broader project about eating behavior and was reviewed and approved by the Ethics Committee of Iscte-Instituto Universitário de Lisboa (approval #22/2019). Instructions stated the goals and expected duration of the study, as well as ethical considerations (i.e., anonymity, confidentiality, and the possibility to withdraw from the study at any point) and conditions to participate (i.e., over 18 years adult, Portuguese nationality). Written informed consent was obtained from all participants, and the only incentive to collaborate was the possibility to enter a raffle to win one of three EUR 50 commercial vouchers. 

**Figure 1:**
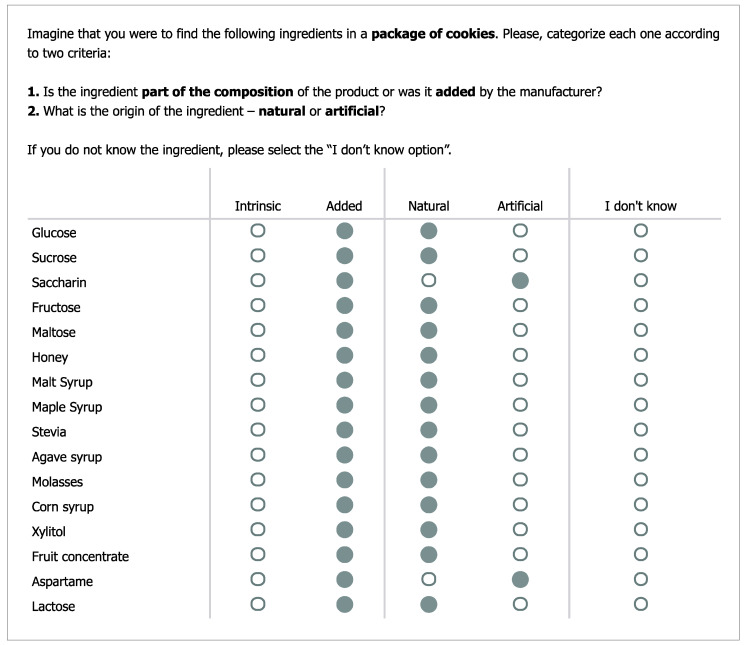
Categorization of sugar sources task (Qualtrics). Sugar sources were presented in random order. The options selected represent the correct answers (hits). Original instructions and items were presented in Portuguese.

Invitation to participate in a web survey (hosted in Qualtrics) about food habits was shared on social networks (e.g., Facebook, LinkedIn) for about two weeks. Participants were asked to provide sociodemographic information, answer a set of questions about general food habits, and then to answer questions regarding knowledge about sugar sources and sugar intake guidelines. The answers were provided in 7-point response scales and were presented in the following order:

1. Frequency of use and perceived importance of nutritional information: To assess the type of nutritional information prioritized by participants, we included two items (adapted from [20]). First, we asked participants to indicate how frequently they take into consideration each of the following items of the nutritional label: calories, total fat, saturated fat, proteins, total carbohydrates, sugar, and salt (from 1 = Never to 7 = Always). Responses were averaged into a single index (*α* = 0.93). Second, we asked participants to indicate how important they believe it is to watch each item to stay healthy (from 1 = Not at all important to 7 = Very important). Again, responses were averaged into a single index (α = 0.89). In both cases, the seven items of the nutritional table were presented in random order.
2. Short Food Literacy Questionnaire (SFLQ): We translated the SFLQ [32] to European Portuguese to assess self-reported food literacy. The original measure included 12 items (e.g., “When I have questions on healthy nutrition, I know where I can find information on this issue”; from 1 = Strongly Disagree to 7 = Strongly Agree). Besides cultural adaptations (e.g., “Swiss Food Pyramid” was replaced by “Food Wheel”), we also standardized the number of points of the rating scales across all items (i.e., 7-point response scales). Moreover, the response options to the item related to sources of nutritional information (“How well do you understand the following types of nutritional information?”) included a new type of source (i.e., scientific papers or books). Finally, the original measure included an item regarding the perceived knowledge of the guidelines for salt intake, and we added a new item regarding the guidelines for sugar intake (“I know the guidelines for sugar intake for the Portuguese population”). After confirming the adequacy of the sampling based on the Kaiser-Meyer-Olkin (KMO) and Bartlett’s test of sphericity (KMO = 0.90), an exploratory factor analysis with principal axis factoring and Promax rotation allowed the extraction of two factors (eigenvalues > 1) that accounted for 58.07% of the variance. Factor 1 (eigenvalue 5.81; 44.67% of explained variance) included nine items (*α* = 0.88) related to the perceived ability of finding, understanding, and assessing nutritional information (SFLQ_1). Factor 2 (eigenvalue 1.74; 13.40% of explained variance) included four items (*α* = 0.87) related to the perceived knowledge of nutritional guidelines (SFLQ_2; for descriptive results and factor loadings per item, see Appendix A).
3. Frequency of consumption of sugary products: To assess the frequency of consumption of sugary products, we included a general item—“Usually, how frequently do you consume high sugar foods and drinks?” (1 = Never or less than once a month; 2 = 1 to 3 times/month, 3 = once a week, 4 = 2 to 4 times/weeks, 5 = 5 to 6 times/week, 6 = once a day, 7 = more than once a day).
4. Knowledge categorization of sugar sources: This task was based on the one used by Tierney et al. [20] with the following exceptions: (a) the list of “sugar” sources included 16 items (e.g., sucrose, honey, aspartame—this set included different types of sugars and sweeteners; for the sake of brevity, we refer to this list as sugar sources), instead of 13; (b) these sources were presented in random order, instead of a fixed order; and (c) the categorization included two criteria—composition (i.e., intrinsic vs. added) and origin (i.e., natural vs. artificial)—instead of a single one (original response options were “natural sugar”, “added/free sugar” and “artificial sweetener”). We have also changed the instructions to emphasize that all sugar sources were ingredients for another food product and not as food products on their own (e.g., honey is a natural product with intrinsic sugars that become a source of added sugars when used to make cookies). The categorization task (including instructions and the full list of 16 items) is presented in Figure 1.
5. Knowledge of the WHO guidelines regarding sugar intake. We stated that the WHO had recently defined guidelines regarding the intake of free sugars and presented the definition of free sugars (we used the official Portuguese definition, see [33]). Then, we asked participants to use 7-point rating scales to indicate their opinion regarding how easy it was to understand that definition (from 1 = It is hard to comprehend what free sugars are to 7 = It is easy to comprehend what free sugars are) and to identify free sugars in products (from 1 = Identifying free sugars in products is hard to 7 = Identifying free sugars in products is easy). The items were strongly and positively correlated, *r* = 0.75, *p* < 0.001. Next, we asked participants to indicate whether they knew the maximum amount of sugar recommended and provided three response options (1 = The daily intake of sugar should be limited to___ g, 2 = I don’t know about these guidelines, 3 = I have heard about these guidelines, but I can’t recall the exact value).
  In the last block of questions, participants were asked to indicate their type(s) of diet(s), height, and weight (open-ended answers, including “I don’t know/I rather not say” options). Lastly, participants were thanked and debriefed.

## 3. Results

### 3.1. Analytical Plan

All the questionnaires were retained for analysis (*n* = 1010). First, we characterize the overall frequency of use and perceived importance of nutritional information and examine whether these indicators vary according to the specific type of nutritional information (e.g., sugar, salt, or calories, Section 3.2). Next, we present descriptive results regarding the accuracy in the categorization of sugar sources (Section 3.3), and self-reported food literacy (SFLQ), knowledge of the WHO guidelines, and ease of comprehension and identification of free sugars (Section 3.4). Subsequently, we examine the role played by individual characteristics—gender, having children in the household, and level of education—across variables (Section 3.5), as well as correlations between the main variables (Section 3.6). Finally, we present results from a linear hierarchical regression examining how individual and sugar consumption-related variables shape performance in the task of categorizing sugar sources (Section 3.7).

### 3.2. Frequency of Use and Perceived Importance of Nutritional Information

**Figure 2:**
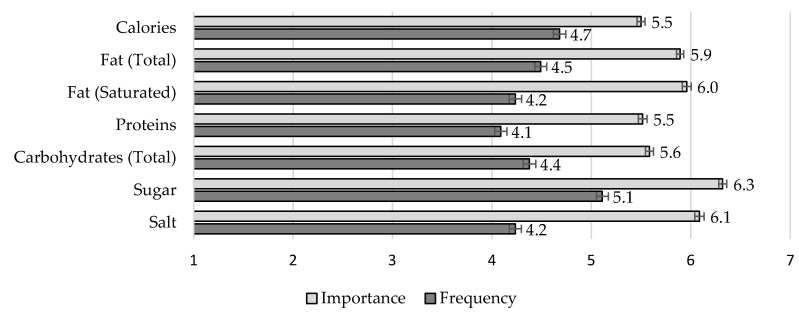
Frequency of use and perceived importance according to the type of nutritional information.

Each variable was analyzed with a one-way ANOVA with repeated measures (results with Huynh–Feldt correction as the sphericity assumption was not verified). Mean results are presented in Figure 2. Overall, participants reported using nutritional information frequently (*M* = 4.46, *SE* = 0.051, 95% CI [4.36, 4.56]). Still, we observed a main effect of type of nutritional information, *F*(5.16, 5204.70) = 90.27, *MSE* = 140.55, *p* < 0.001, *η^2^_p_* = 0.082. Pairwise comparisons with Bonferroni correction showed that sugar was the most frequently used type of nutritional information, all *p*s < 0.001, and proteins were the least frequently used, all *p*s < 0.001, but not different from salt, *p* = 0.236, and saturated fat, *p* = 0.102.

Overall, participants also reported that using nutritional information was important to stay healthy (*M* = 5.84, *SE* = 0.032, 95% CI [5.77, 5.91]). Importance ratings also varied according to the type of nutritional information, *F*(4.76, 4785.07) = 121.79, *MSE* = 125.75, *p* < 0.001, *η^2^_p_* = 0.108. Again, sugar was deemed the most important nutrient to attend to in order to stay healthy, all *p*s < 0.001. In contrast, both caloric content and proteins were deemed the least important nutrients to attend to, all *p*s < 0.001, but not different from total carbs, *p* = 0.909.

### 3.3. Knowledge: Categorization of Sugar Sources

**Figure 3:**
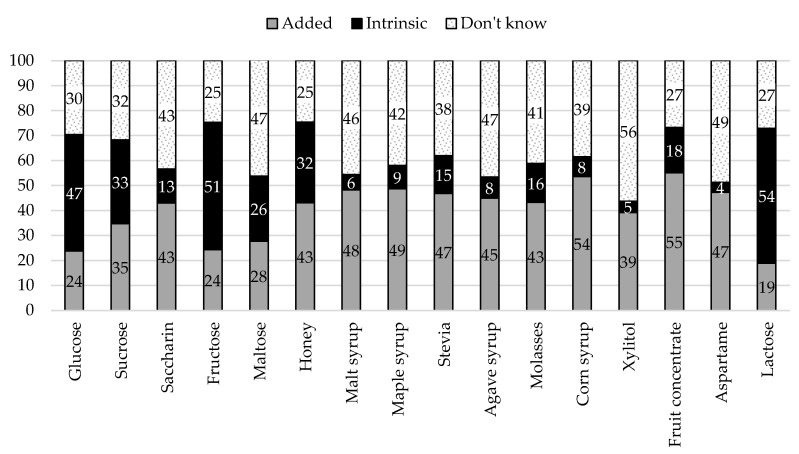
Categorization of sugar sources according to composition.

**Figure 4:**
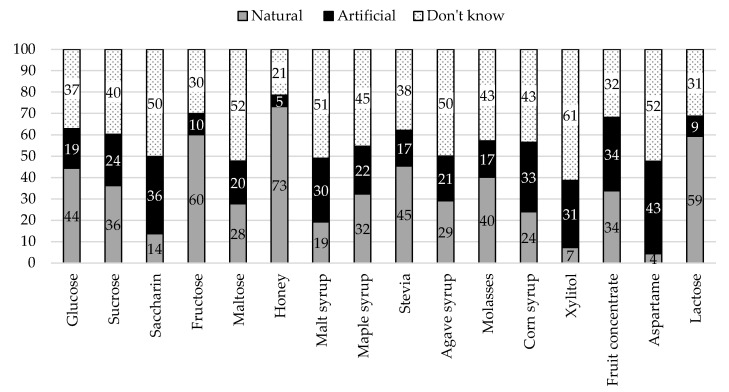
Categorization of sugar sources according to origin.

For each sugar source, we calculated frequencies (and percentages) according to each criterion—composition (i.e., Added vs. Intrinsic sugar) and origin (i.e., Natural vs. Artificial). Overall, participants showed low accuracy in categorizing sugar sources according to composition (*M* = 42.80, *SD* = 30.62, 95% CI [40.91, 44.69]) and origin (*M* = 39.86, *SD* = 26.22, 95% CI [38.24, 41.48]). Results per sugar source are summarized in Figure 3 and Figure 4.

According to the scenario presented (i.e., list of ingredients of packaged cookies), all items were sources of added sugar. Therefore, “intrinsic” responses correspond to errors in categorization. As shown in Figure 3, the items more commonly misclassified as intrinsic sources of sugar were lactose (54%), fructose (51%), and glucose (47%). It also noteworthy the high percentage of “I don’t know” (or missing) responses that varied from 25% for fructose and honey up to 56% for xylitol.

All items referred to natural sugar sources, except aspartame and saccharine. Indeed, these were the items with the highest percentages of “artificial” responses (43% and 36%, respectively). Still, participants often incorrectly categorized other sugar sources as artificial, namely fruit concentrate (34%), corn syrup (33%), or xylitol (31%).

### 3.4. Short Food Literacy Questionnaire (SFLQ), Knowledge of WHO Guidelines and Ease of Comprehension and Identification of Free Sugars

Overall, participants reported having an above-average capability of finding, understanding and assessing nutritional information (SFLQ_Factor 1; *M* = 4.92, *SD* = 1.04, 95% CI [4.86, 4.98]), as well as adequate knowledge of nutritional guidelines (SFLQ_Factor 2; *M* = 5.78, *SD* = 1.19, 95% CI [4.86, 4.98]).

Moreover, participants reported above-average (*M* = 4.15, *SD* = 1.95, 95% CI [4.03, 4.27]) ability to comprehend what free sugars are. However, the ease of identification of these sugars in products was below average (*M* = 3.81, *SD* = 1.90, IC 95% [3.69, 3.92]).

When asked about the guidelines, most participants (38.0%) responded that they did not know about the WHO guidelines regarding sugar intake or heard about it but did not remember the exact value (35.8%). In total, 264 (26.1%) participants selected the option to provide an estimate of the recommended sugar intake. However, eight failed to provide the estimate (e.g., answers “I don’t know” or “it depends on the person”). Whenever necessary, the estimates were recoded (e.g., “between 30 to 40 g” recoded as “35 g”; and “ideally no sugar at all” recoded as “0”). Extreme outliers (*n* = 11, estimates ≥ 100 g/day) were recoded as missing values. Participants indicated an estimate of the maximum intake of free sugar (g/day) that varied between 0 and 96 (*M* = 24.91*; SD* = 18.35). This estimate did not vary according to gender, *t* < 1, having higher education, *t*(243) = 1.44, *p* = 0.151, or children in the household, *t*(243) = 1.28, *p* = 0.203).

### 3.5. Individual Differences

Table 1 systematizes differences across variables according to gender, having children in the household, and education level.

#### 3.5.1. Gender

As shown in Table 1, women reported using all types of nutritional information more frequently than men, all *p*s ≤ 0.002, except protein content, *p* = 0.122. The overall pattern was similar for perceived importance, with women indicating higher ratings of perceived importance for all types of nutrition information, all *p*s ≤ 0.032, except calories, *p* = 0.952, and proteins, *p* = 0.060. In addition, women (vs. men) obtained higher hit rates in both criteria of the categorization of sugar sources, that is, composition, *p* < 0.001, and origin, *p* = 0.004. 

Likewise, concerning self-reported nutritional literacy, women (vs. men) reported both higher perceived capability and higher perceived knowledge of nutritional guidelines, *p*s < 0.001. Finally, women reported finding it easier to comprehend the definition of free sugars than men, *p* = 0.013. Still, no gender differences emerged for the ease of identification of free sugars in products, *t* < 1, nor for the index that includes both these measures, *p* = 0.087.

#### 3.5.2. Children in the Household

In general, as shown in Table 1, we did not observe differences in ratings according to the presence of children in the household. Still, those with (vs. without) children reported using information regarding sugar, *p* = 0.029, and salt, *p* = 0.004, more often (all other *p*s ≥ 0.085). No differences according to having children in the household were detected for perceived importance of nutritional information, all *p*s ≥ 0.059. 

Moreover, participants with (vs. without) children in the household were more accurate in identifying added versus intrinsic sugars than those without children, *p* = 0.023. This difference did not emerge in their accuracy for the origin criterion, *p* = 0.186. As for self-reported nutritional knowledge, we found that participants with (vs. without) children reported higher perceived knowledge of nutritional guidelines, *p* = 0.001, but not higher perceived capability to deal with nutritional information, *p* = 0.425. Finally, no difference was observed according to having children in the reported ease of comprehension of free sugar definition or ease of identification of these sugars in products, *p* ≥ 0.421.

#### 3.5.3. Level of Education

We found differences in ratings according to education level (see Table 1), namely: participants with (vs. without) higher education reported using information related to calories, *p* = 0.014, total carbohydrates, *p* = 0.017, and sugar, *p* = 0.012, more frequently. The pattern was slightly different for perceived importance of nutritional information: participants with (vs. without) higher education attributed higher importance to saturated fat, *p* = 0.027, sugar, *p* < 0.001, and salt, *p* = 0.029.

Additionally, higher hit rates were obtained for participants with (vs. without) higher education for both criteria of the categorization task (i.e., composition and origin), *p*s < 0.001.

Participants with (vs. without) higher education reported higher capability to deal with nutritional information, *p* = 0.002, but not higher knowledge of nutritional guidelines, *p* = 0.057. Finally, participants with (vs. without) higher education reported finding it easier to comprehend the definition of free sugars, *p* = 0.013, but no differences were found for the ease of identification of free sugars in products, *p* = 0.880.

### 3.6. Correlations

Results regarding the correlation between variables are presented in Table 2. Hit rates regarding the categorization of sugar sources composition and origin were positively correlated. These hit rates were also positively correlated with the frequency of using sugar content information and the perceived importance of attending to sugar information to stay healthy, with both SFLQ factors, and with perceived ease of comprehending and identifying free sugars. Moreover, participants who reported using sugar information more frequently also considered more important attending to sugar information in order to stay healthy, scored higher on food literacy, and found it easier to comprehend and identify free sugars. The importance attributed to sugar in order to stay healthy was also positively associated with both SFLQ factors, which were positively associated with the ease of comprehending and identifying free sugars. 

Age was only negatively associated with hits rates in categorizing sugar sources composition and positively associated with perceived knowledge about nutritional guidelines (SFLQ_2). BMI was also negatively associated with hit rates regarding the categorization of sugar sources composition. Finally, self-reported frequency of sugar consumption was negatively associated with all variables (except BMI).

### 3.7. Hierarchical Regression Analysis

Table 3 presents the results of the hierarchical regression with accuracy (i.e., overall hits) in the sugar sources categorization task as the outcome variable. We have also conducted this analysis for each criterion (composition and origin) separately. Because the pattern of results was similar, for the sake of clarity, we choose to present results for an aggregated accuracy measure. In each block, we entered individual characteristics (Step 1), the general frequency of high-sugar products consumption (Step 2), and variables related to knowledge about sugar (Step 3). The final model was significant, *F*(11, 935) = 19.07, *MSE* = 9386.16, *p* < 0.001, and explained 17% of the variance in the overall accuracy categorization of sugar sources task. 

As shown in Table 3, results of Step 1 indicate that general accuracy in the categorization task was significantly associated with gender, *p* = 0.001, children in the household, *p* = 0.005, and education, *p* < 0.001, such that higher accuracy was found for women, those with children and with higher education. The inclusion of frequency of sugar consumption in Step 2 was also significant, *p* = 0.008, and did not change the associations with these individual characteristics. Still, when variables related to knowledge regarding sugar were introduced in Step 3, gender differences became non-significant, *p* = 0.320, but the role of having children and level of education remained significant, *p* = 0.005 and *p* < 0.001, respectively. The strongest associations were found for the frequency of looking at sugar information in nutritional labels, *p* < 0.001, and for the ease of reported comprehension and identification of free sugars, *p* = 0.002. The perceived importance of taking sugar into consideration as a strategy to stay healthy was also positively associated with the accuracy in categorizing sugar sources, *p* = 0.044. Finally, regarding the factors of self-reported food literacy, we observed a positive association between the perceived capability factor and accuracy, *p* = 0.042.

## 4. Discussion

The negative health implications of excessive sugar consumption led the WHO, in 2015, to issue sugar intake guidelines to address this problem. Despite the sugar reduction strategies that have been adopted in several countries, namely in Portugal (for a review, see [31]), sugar intake is still high. For example, almost one-fourth of the adult Portuguese population consumes free sugars in quantities higher than those recommended by the WHO [29]. In this study, we aimed to characterize the knowledge of a sample of Portuguese consumers about sugar sources and guidelines for sugar intake and examine whether that knowledge was associated with other variables related to the ability to process nutritional information. Moreover, we also examined how individual characteristics shape these variables.

Overall, our results showed that participants value information about sugar content and report that sugar is the item in the nutrition panel they attend to more frequently. Congruently, they also consider that sugar content is the most important item to attend to in order to stay healthy. Results from the self-report measure of food literacy [32] revealed that, overall, participants considered having a high capability of dealing with nutritional information and adequate knowledge of nutritional guidelines. Still, when asked to categorize sugar sources and indicate the WHO sugar intake guidelines, the results were not optimistic. When presented with the official free sugars definition, participants indicated that comprehending it is somewhat easy, but identifying free sugars in products is hard.

Moreover, as in previous studies [20,21], most participants reported not being aware or not remembering the WHO guidelines, and only about one-fourth of the sample provided an accurate estimate of the maximum free sugar intake recommended. On average, these participants stated that sugar should be limited to 25 g/day, which would correspond to the most conservative [1] recommendation. However, because the range varied between 0 and 96 g/day, it is not possible to ascertain that they indeed know the guidelines. 

Regarding the ability to categorize the sources of sugar, results suggest that participants actually recognize their lack of knowledge. For instance, percentages of “*I don’t know*” answers varied between 25 and 56% for the composition criterion and between 21 and 61% for the origin criterion. In both criteria, the items that seemed to raise fewer doubts (i.e., fewer “*I don’t know*” answers) were honey and fructose, whereas the highest uncertainty was found for xylitol and aspartame. This task is challenging as added sugars are usually referred to on nutritional labels by unfamiliar names, which participants may not recognize [34,35]. The inclusion of these two criteria (composition and origin) in the task developed by Tierney et al. [20] aimed to reduce the influence of a potential bias (i.e., the association that something natural is also an intrinsic source of sugar). For example, in the original study, the authors reported very high error rates for ingredients such as honey (i.e., not identified as added sugar by 89% of the sample). In contrast, we observed that most of our participants indicated that although honey is of natural origin (73%), when included as an ingredient in processed foods it is actually a source of added sugar (43%). Still, items most often recognized as natural (e.g., lactose and fructose) were often miscategorized as intrinsic sugars. In addition, results regarding the origin criterion can inform us about consumers’ receptivity to certain ingredients, as consumers tend to mistrust food additives, viewing them as less natural and healthy [36]. For example, although a moderate consumption of xylitol has been associated with various health benefits [37], a high percentage of our participants categorized it as artificial, which can hinder the choice for products with this sweetener. Moreover, the categorization task was positively associated with some sociodemographic characteristics (particularly level of education and the presence of children in the household) as well as variables such as the frequency of looking at sugar information in nutritional labels and the reported ease of comprehension and identification of free sugars.

We observed differences in several measures according to sociodemographic characteristics such that, overall, women, participants with higher education, and with children in the household performed better. For example, we observed that women (vs. men) report using nutritional information more often and perceive it as more important to stay healthy. Although Tierney et al. [20] did not find such differences regarding the use of information available on the nutrition labels, several studies found a pattern similar to our study [38]. These studies report that men care less about the information on nutrition labels, a result explained by the fact that they are less interested in topics related to health and food in general [39,40]. Indeed, women seem to be more health-conscious than men [39,40] and more able to make healthier decisions based on nutritional information [41,42]. The fact that men use less nutritional information can also be explained by the traditional social role of women being responsible for buying the food and preparing the family’s meals [34,35].

Additionally, our results revealed that women found it easier to comprehend the definition of free sugars and were more accurate in identifying whether a sugar source was added or intrinsic to the product or of natural versus artificial origin. This result was not found by Tierney et al. [20] but is consistent with other findings indicating that women are more aware of nutritional issues, which provides them with better nutritional knowledge [43]. Unsurprisingly, people with a higher (vs. lower) level of education reported to use nutritional information more often and perceive it as more important (e.g., [44,45]). Additionally, they were more accurate in identifying the composition and origin of sugar sources and considered the definition of free sugars easier to comprehend. These results are in line with past findings showing that a high level of education is associated with a higher nutritional knowledge (e.g., individuals with higher education have more access to nutritional information (for a review, see [32]). Indeed, the difficulty in interpreting nutritional labels is particularly critical in less-educated people [30].

We also found some interesting differences regarding the presence of children in the household. For example, those with (vs. without) children in the household reported using information regarding sugar (and salt) content more often and were more accurate in identifying added sugars (vs. intrinsic sugars). This result is very important, given that parents play an important role in developing their children’s eating habits [20,46]. Additionally, 11.7% of Portuguese children between 6 to 8 years old are obese, and 30.7% are overweight [33]. It would be interesting to examine in future studies if being more attentive to sugar content and nutritional labels and being more knowledgeable about sugar sources has an impact on foods made available for the children in the household and, ultimately, on their weight. 

One main limitation of the present study is that our sample has a higher proportion of women and of people with higher education than the Portuguese population, variables that have been associated with higher levels of nutrition knowledge [34,42,47,48,49]. Therefore, these results should not be taken as representative of the Portuguese population. Moreover, the method used to assess participants’ knowledge about sugar sources may also present some challenges to the study’s ecological validity, as we asked participants to categorize each ingredient in the absence of a broader context. Notably, categorizing sugars in real-life settings is expected to be more demanding due to the myriad of information presented in food packaging. For example, besides the nutrition information panel, the ingredients list, and branding features, food packages often include various claims about the origin, production method, composition, or health and nutritional benefits of the products. These aspects could be explored in future studies by using more naturalistic materials (e.g., categorizing sugar sources of existing food products based on the information available in the package). Importantly, the present study did not analyze whether individuals adhere to the WHO guidelines and recommendations for reducing sugar intake, but only their knowledge about these guidelines. Knowledge itself may not be enough to reduce sugar intake [20,46]. Future studies could assess barriers and facilitators of compliance with these recommendations, as it was done for other dietary guidelines (e.g., fruit and vegetables consumption, [50,51,52]. 

This study is one of the few to examine knowledge about sugar sources and guidelines to reduce sugar intake (see also, [20,53,54]) and, to the best of our knowledge, the first one to be conducted with a Portuguese sample. Most participants in this study experienced difficulties in categorizing sugar sources and seemed to be unaware of the WHO’s guidelines for reducing sugar intake. Our findings also indicate that despite the recent strategies adopted in Portugal to address excessive sugar intake (e.g., increased taxes for more sugary foods, [31]) and its consequences for people’s health (e.g., diabetes), information about sugar sources and intake guidelines may still be insufficient. Taken together, our findings emphasize the need to develop further strategies and measures to improve nutritional literacy in general and knowledge about sugar in particular. For instance, simplifying information and methods of presenting nutritional information on labels can be an instrumental strategy for improving healthy food choices and to counteract the harmful effects of excessive sugar intake [55]. Moreover, our results suggest that some target groups may benefit particularly from interventions aimed at developing knowledge about sugar (e.g., people with lower education levels). These interventions may be based on evidence regarding the effectiveness of strategies to control other behaviors with negative health effects (e.g., smoking, for a review see [56]).

## Figures and Tables

**Table 1:** Individual differences across variables according to gender, presence of children in the household, and level of education.

	Gender				Children in the Household				Higher Education			
	Men (*n* = 231)		Women (*n* = 775)		No (*n* = 672)		Yes (*n* = 338)		No (*n* = 215)		Yes (*n* = 795)	
	*M*	(*SD*)	*M*	(*SD*)	*M*	(*SD*)	*M*	(*SD*)	*M*	(*SD*)	*M*	(*SD*)
Frequency of use												
Calories	4.18 ^a^	(2.01)	4.83 ^b^	(1.87)	4.66 ^c^	(1.98)	4.71 ^c^	(1.81)	4.40 ^e^	(2.08)	4.76 ^f^	(1.87)
Fat (Total)	4.02 ^a^	(2.01)	4.63 ^b^	(1.89)	4.42 ^c^	(2.03)	4.64 ^c^	(1.73)	4.27 ^e^	(2.08	4.55 ^e^	(1.89)
Fat (Saturated)	3.84 ^a^	(2.00)	4.35 ^b^	(1.99)	4.18 ^c^	(2.08)	4.35 ^c^	(1.86)	4.08 ^e^	(2.11)	4.28 ^e^	(1.98)
Proteins	3.91 ^a^	(2.01)	4.14 ^a^	(1.93)	4.08 ^c^	(2.02)	4.10 ^c^	(1.81)	3.88 ^e^	(2.00)	4.14 ^e^	(1.94)
Carbohydrates (Total)	4.00 ^a^	(2.02)	4.49 ^b^	(1.93)	4.36 ^c^	(2.03)	4.42 ^c^	(1.81)	4.09 ^e^	(2.05)	4.45 ^f^	(1.93)
Sugar	4.42 ^a^	(2.04)	5.31 ^b^	(1.76)	5.02 ^c^	(1.93)	5.29 ^d^	(1.71)	4.83 ^e^	(2.00)	5.19 ^f^	(1.82)
Salt	3.88 ^a^	(2.03)	4.34 ^b^	(1.97)	4.11 ^c^	(2.03)	4.49 ^d^	(1.88)	4.17 ^e^	(2.06)	4.25 ^e^	(1.97)
Total	4.04 ^a^	(1.79)	4.58 ^b^	(1.56)	4.40 ^c^	(1.70)	4.57 ^c^	(1.45)	4.25 ^e^	(1.77)	4.52 ^f^	(1.58)
Perceived Importance ^1^												
Calories	5.52 ^a^	(1.46)	5.51 ^a^	(1.50)	5.52 ^c^	(1.51)	5.47 ^c^	(1.45)	5.54 ^e^	(1.57)	5.49 ^e^	(1.47)
Fat (Total)	5.60 ^a^	(1.40)	5.98 ^b^	(1.23)	5.92 ^c^	(1.28)	5.83 ^c^	(1.27)	5.77 ^e^	(1.43)	5.92 ^e^	(1.23)
Fat (Saturated)	5.69 ^a^	(1.40)	6.04 ^b^	(1.26)	6.00 ^c^	(1.31)	5.89 ^c^	(1.28)	5.79 ^e^	(1.48)	6.01 ^f^	(1.25)
Proteins	5.37 ^a^	(1.41)	5.56 ^a^	(1.38)	5.58 ^c^	(1.37)	5.40 ^c^	(1.43)	5.54 ^e^	(1.41)	5.51 ^e^	(1.38)
Carbohydrates (Total)	5.42 ^a^	(1.42)	5.64 ^b^	(1.37)	5.60 ^c^	(1.41)	5.55 ^c^	(1.34)	5.54 ^e^	(1.44)	5.60 ^e^	(1.37)
Sugar	6.04 ^a^	(1.31)	6.40 ^b^	(1.08)	6.32 ^c^	(1.15)	6.32 ^c^	(1.14)	6.06 ^e^	(1.44)	6.38 ^f^	(1.04)
Salt	5.77 ^a^	(1.43)	6.18 ^b^	(1.22)	6.07 ^c^	(1.31)	6.12 ^c^	(1.22)	5.92 ^e^	(1.53)	6.14 ^f^	(1.20)
Total	5.63 ^a^	(1.16)	5.90 ^b^	(0.98)	5.86 ^c^	(1.03)	5.80 ^c^	(1.03)	5.74 ^e^	(1.18)	5.87 ^e^	(0.98)
Sugar sources categorization												
1. Hits: Composition	36.04 ^a^	(28.94)	44.88 ^b^	(30.84)	41.24 ^c^	(30.51)	45.90 ^d^	(30.65)	31.34 ^e^	(30.63)	45.90 ^f^	(29.89)
2. Hits: Origin	35.52 ^a^	(25.15)	41.11 ^b^	(26.34)	39.09 ^c^	(26.49)	41.40 ^c^	(25.64)	28.66 ^e^	(25.68)	42.89 ^f^	(25.55)
SFLQ ^1^												
Perceived capability (SFLQ_1)	4.68 ^a^	(1.03)	4.99 ^b^	(1.03)	4.90 ^c^	(1.06)	4.96 ^c^	(1.01)	4.72 ^e^	(1.06)	4.97 ^f^	(1.03)
Perceived knowledge (SFLQ_2)	5.44 ^a^	(1.35)	5.88 ^b^	(1.12)	5.69 ^c^	(1.23)	5.96 ^d^	(1.08)	5.64 ^e^	(1.22)	5.82 ^e^	(1.18)
Ease of comprehension of FS ^2^ definition	3.88 ^a^	(1.81)	4.24 ^b^	(1.98)	4.13 ^c^	(1.95)	4.20 ^c^	(1.96)	3.90 ^e^	(1.99)	4.22 ^f^	(1.93)
Ease of identification of FS	3.74 ^a^	(1.82)	3.83 ^a^	(1.91)	3.77 ^c^	(1.92)	3.87 ^c^	(1.85)	3.82 ^e^	(1.93)	3.80 ^e^	(1.89)
Total	3.81 ^a^	(1.72)	4.04 ^a^	(1.82)	3.95 ^c^	(1.80)	4.03 ^c^	(1.79)	3.86 ^e^	(1.85)	4.00 ^e^	(1.79)

Note. Different superscripts indicate differences (independent samples *t-*tests) according to gender (^a,b^), presence of children in the household (^c,d^), or education level (^e,f^). ^1^ SFLQ—Short Food Literacy Questionnaire; SFLQ_1—SFLQ Factor 1: Perceived capability finding, understanding, and assessing nutritional information; SFLQ_2—SFLQ Factor 2: Perceived knowledge of nutritional guidelines. ^2^ FS—Free Sugars.

**Table 2:** Correlations.

	1.	2.	3.	4.	5.	6.	7.	8.	9.
1. Hits: Composition	-								
2. Hits: Origin	0.48 ***	-							
3. Sugar: Frequency of use	0.26 ***	0.29 ***	-						
4. Sugar: Importance	0.16 ***	0.16 ***	0.40 ***	-					
5. Perceived capability (SFLQ_1)	0.22 ***	0.21 ***	0.41 ***	0.20 ***	-				
6. Perceived knowledge (SFLQ_2)	0.19 ***	0.13 ***	0.33 ***	0.24 ***	0.52 ***	-			
7. Ease of comprehension and identification FS	0.13 ***	0.16 ***	0.11 **	−0.03	0.41 ***	0.25 ***	-		
8. Age	−0.08 **	−0.06	0.02	−0.04	0.03	0.13 ***	0.00	-	
9. BMI	−0.09 **	−0.04	−0.03	−0.03	−0.06	−0.04	0.00	0.28 ***	-
10. Frequency of sugar consumption	−0.10 **	−0.07 *	−0.25 ***	−0.11 ***	−0.26 ***	−0.18 ***	−0.13 ***	−0.17 ***	0.05

Note. Variables 1 and 2 refer to the mean results observed in the categorization task of the sugar sources for the composition and origin criteria, respectively. Variables 3 and 4 refer to the items regarding the frequency of use of sugar information and the perceived importance of attending to sugar information in order to stay healthy. *** *p* < 0.001, ** *p* < 0.010, * *p* < 0.050.

**Table 3:** Hierarchical regression analysis: accuracy in the sugar categorization task.

	B	SE B	*β*
*Step 1*			
Constant	26.50	5.72	
Gender (women = 1; men = 0)	5.94	1.86	0.10 **
Age	−0.02	0.06	−0.01
Children in the household (1 = yes; no = 0)	4.55	1.62	0.09 **
Education (superior = 1 vs. not superior = 0)	13.91	1.97	0.23 ***
BMI	−0.06	0.19	−0.01
*Step 2*			
Constant	31.96	6.06	
Gender (women = 1; men = 0)	5.78	1.86	0.10 **
Age	−0.06	0.06	−0.03
Children in the household (1 = yes; no = 0)	4.50	1.62	0.09 **
Education (superior = 1 vs. not superior = 0)	13.23	1.98	0.22 ***
BMI	−0.02	0.19	0.00
Frequency of sugar intake	−1.36	0.51	−0.09 **
*Step 3*			
Constant	−7.54	7.71	
Gender (women = 1; men = 0)	1.79	1.80	0.03
Age	−0.07	0.06	−0.04
Children in the household (1 = yes; no = 0)	3.52	1.55	0.07 *
Education (superior = 1 vs. not superior = 0)	11.68	1.89	0.19 ***
BMI	−0.07	0.18	−0.01
Frequency of sugar intake	0.14	0.50	0.01
Sugar: Frequency of use	2.79	0.47	0.21 ***
Sugar: Importance	1.41	0.70	0.07 *
Perceived capability (SFLQ_1)	1.87	0.91	0.08 *
Perceived knowledge guidelines (SFLQ_2)	0.50	0.74	0.03
Ease of comprehension and identification FS	1.40	0.45	0.10 **

Note*. R*^2^ = 0.17. *** *p* < 0.001, ** *p* < 0.010, * *p* < 0.050.

## Appendix A

Descriptive Results (*M*, *SD*) and Factor Loadings for the SFLQ.

	***M***	***SD***	**Factor Loading ^a^**
Factor 1 (SFLQ_1): Perceived capability finding, understanding, and assessing nutritional information			
1. When I have questions on healthy nutrition, I know where I can find information on this issue.	5.24	1.48	0.57
2. In general, how well do you understand the following types of nutritional information? (6 sources)	4.82	1.29	0.48
7. Think about a usual day: how easy or difficult is it for you to compose a balanced meal at home?	5.24	1.47	0.48
8. In the past, how often were you able to help your family members or a friend if they had questions concerning nutritional issues?	4.09	1.74	0.55
9. There is a lot of information available on healthy nutrition today. How well do you manage to choose the information relevant to you?	5.20	1.39	0.77
10. How easy is it for you to judge if media information on nutritional issues can be trusted?	4.47	1.57	0.84
11. Commercials often relate foods with health. How easy is it for you to judge if the presented associations are appropriate or not?	4.55	1.57	0.80
12. How easy is it for you to evaluate if a specific food is relevant for a healthy diet?	5.34	1.27	0.75
13. How easy is it for you to evaluate the longer-term impact of your dietary habits on your health?	5.35	1.39	0.60
Factor 1 Total	4.92	1.04	-
Factor 2 (SFLQ_2): Perceived knowledge of nutritional guidelines			
3. How familiar are you with the Food Wheel?	5.91	1.18	0.41
4. I know the recommendations about fruit and vegetable consumption for the Portuguese population.	5.71	1.46	0.77
5. I know the recommendations about salt intake for the Portuguese population.	5.70	1.49	0.93
6. I know the recommendations about sugar intake for the Portuguese population.	5.79	1.47	0.92
Factor 2 Total	5.78	1.19	-
Note. Ratings for all items varied between 1 and 7. Sample size for all items = 1010, except for Item 2 (*n* = 993). ^a^ Principal axis factoring analysis with Promax rotation.			
